# An inducible rodent glaucoma model that exhibits gradual sustained increase in intraocular pressure with distinct inner retina and optic nerve inflammation

**DOI:** 10.1038/s41598-021-02057-w

**Published:** 2021-11-24

**Authors:** David J. Mathew, Izhar Livne-Bar, Jeremy M. Sivak

**Affiliations:** 1 Donald K. Johnson Eye Institute, Krembil Research Institute, University Health Network, Toronto, ON Canada; 2 Department of Ophthalmology and Vision Sciences, University of Toronto, Toronto, ON Canada; 3 Department of Lab Medicine and Pathobiology, University of Toronto, Toronto, ON Canada

**Keywords:** Biological techniques, Cell death in the nervous system, Visual system

## Abstract

Glaucoma is a chronic and progressive neurodegenerative disease of the optic nerve resulting in loss of retinal ganglion cells (RGCs) and vision. The most prominent glaucoma risk factor is increased intraocular pressure (IOP), and most models focus on reproducing this aspect to study disease mechanisms and targets. Yet, current models result in IOP profiles that often do not resemble clinical glaucoma. Here we introduce a new model that results in a gradual and sustained IOP increase over time. This approach modifies a circumlimbal suture method, taking care to make the sutures ‘snug’ instead of tight, without inducing an initial IOP spike. This approach did not immediately affect IOPs, but generated gradual ocular hypertension (gOHT) as the sutures tighten over time, in comparison to loosely sutured control eyes (CON), resulting in an average 12.6 mmHg increase in IOP at 17 weeks (p < 0.001). Corresponding characterization revealed relevant retinal and optic nerve pathology, such as thinning of the retinal nerve fiber layer, decreased optokinetic response, RGC loss, and optic nerve head remodeling. Yet, angles remained open, with no evidence of inflammation. Corresponding biochemical profiling indicated significant increases in TGF-β2 and 3, and IL-1 family cytokines in gOHT optic nerve tissues compared to CON, with accompanying microglial reactivity, consistent with active tissue injury and repair mechanisms. Remarkably, this signature was absent from optic nerves following acute ocular hypertension (aOHT) associated with intentionally tightened sutures, although the resulting RGC loss was similar in both methods. These results suggest that the pattern of IOP change has an important impact on underlying pathophysiology.

## Introduction

Glaucoma is a chronic and progressive neurodegenerative disease of the optic nerve resulting in permanent loss of retinal ganglion cells (RGCs) and loss of vision. It is a leading cause of irreversible visual impairment and blindness worldwide^1–3^. However, the full pathophysiology of this disease remains unclear^4^. An important contributing factor to this ongoing challenge has been the continuous search for models with improved accuracy to resemble more closely the disease condition being studied. The most prominent glaucoma risk factor is increased intraocular pressure (IOP), and most models focus on reproducing this aspect of the disease^5–7^.

Inducible models offer a rapid and consistent onset of increased IOP in wild type animals. Inducible models that cause ocular hypertension include; laser-induced ocular hypertension, in which the trabecular meshwork or episcleral or limbal blood vessels are targeted^8,9^, hypertonic saline injection into episcleral veins^10,11^, cauterization of episcleral veins^12,13^, injection of hyaluronic acid^14^, microspheres^15^, magnetic^16^ or non-magnetic microbeads^17^, and via circumlimbal suture^18–20^. Other inducible models that do not result in ocular hypertension include optic nerve transection or crush^21,22^, intravitreal injection of excitotoxic agents that target glutamate receptors^23–27^ and ischemia–reperfusion injury^28–30^. However, existing inducible models still have substantial limitations, such as; a severe spike in intraocular pressure (IOP) at induction followed by a slow decline that does not resemble the gradual increase observed in clinical primary open angle glaucoma, intraocular entry and potential inflammation, and the need for multiple interventions to maintain elevated IOP over a longer duration. Consequently, it remains unclear how these differences impact the resulting pathological mechanisms and interpretations. In particular, the pathological impact of acute vs gradual IOP profiles remains poorly defined. Thus, there is a continued need for a model that overcomes these limitations.

Here, we report a novel induced model of glaucoma, generated by refining a circumlimbal suture approach. The established circumlimbal suture model involves tightening a Nylon 8-0 suture around the eye, resulting in sustained elevated IOP for 15 weeks^18^. This model has additional advantages of minimal invasiveness, minimal inflammation and reversibility^18,19,31,32^. However, as with other approaches, this model is associated with a sudden acute spike of ocular hypertension (aOHT) at induction followed by a gradual decline, which is not representative of clinical glaucoma. However, evidence suggests ischemic injury may be unlikely if the IOP spike is less than 55 mmHg^33^. When practising this technique, we had serendipitously left some sutures intended to remain ‘loose’ as controls without a tightened IOP spike and noticed that some of these loose sutures subsequently tightened to induce a gradual pressure increase over time. Therefore, we hypothesized that a series of ‘snug’ sutures could be placed to intentionally tighten gradually to exhibit a corresponding gradual increase in ocular hypertension (gOHT) and result in pathophysiology that more closely resembles clinical disease. Therefore, we designed the present experiments to test this gOHT strategy, characterize the resulting retinal and visual pathology, and explore the mechanism underlying this effect. We also took the unique opportunity afforded by this approach to directly compare the effect of aOHT with gOHT for the first time using otherwise similar techniques. Remarkably, although both approaches result in similar RGC loss, biochemical signaling from snug versus tightened sutures reveal significant differences in retinal and optic nerve pathophysiology associated with their distinct IOP profiles.

## Materials and methods

### Circumlimbal suturing technique

All procedures and protocols conformed to the guidelines of the ARVO statement for the use of animals in ophthalmic and vision research and approved by the University Health Network Animal Care and Use Committee. All procedures were performed in accordance with all relevant regulations, and are reported in accordance with ARRIVE guidelines. For the main experiment, 6-week-old Long Evans rats (Charles River Laboratories, Massachusetts, USA) were anesthetized using intraperitoneal Ketamine-Xylazine cocktail. A Nylon 8-0 suture on a tapered needle (8-0 sterile microsuture, AROSurgical Instruments, California, USA) was passed subconjunctivally 1.5 mm posterior to the limbus. The suture was passed all around using 5–6 subconjunctival passes and tied off using a slipknot anchored with 3 simple throws. Adequate caution was exercised to not penetrate the sclera while suturing. For gOHT: Care was taken to do a suturing that was snug, ie; with sutures lying flat against the conjunctiva, but not constricting the eye or tightened as a purse string. A useful practical demonstration when teaching this method was that one arm of a forceps could be easily inserted between the suture and the conjunctiva (see demonstration in Supplementary Video 1). Successful completion of this procedure was verified quantitatively by tonometry measurements at two minutes following the suturing procedure, showing no induced IOP spike for the ‘snug’ method, as compared to the strong IOP spike observed with previously described ‘tightened’ suture method (Supplementary Fig. 1). In comparison, control eyes were sutured very loosely with visible gaps between the conjunctiva and suture passes. For aOHT: Acute sustained increases in ocular hypertension were generated using the previously published ‘tightened’ suture approach (described in detail in^18–20^). In this case the type and placement of the sutures and anchor points followed the same manner described above. However, in this case the suture was firmly tightened until there was gentle, but noticeable resistance from the constricted eye (ie: preventing the easy passage of a forceps arm). The immediate strong elevation of IOP was confirmed by tonometry at two minutes following the procedure (Supplementary Fig. 1). Throughout the experiments, both eyes of each animal were subjected to the same treatment, i.e.; both eyes were either snugly or loosely sutured, in order to avoid potential confounding contralateral effects.

### Intraocular pressure measurement

A Tonolab rebound tonometer (Icare, Finland) was used to measure the IOP according to the manufacturer’s directions. Care was taken to align the tonometer tip perpendicular to the central cornea while obtaining the measurements. Baseline measurements were obtained prior to suturing, after 1 week of alternate day measurements to familiarize the animal to the IOP measurement procedure. IOP measurements were performed at 2 min post suturing, at week 1, and then weekly thereafter. All IOP measurements except the 2-min post-suturing reading were taken while the animal was awake between 10 am and 12 pm. During the IOP measurement, care was taken not to stress the animal or exert pressure on the periocular region. Each measurement with the Tonolab rebound tonometer itself consists of six separate readings, of which the highest and lowest are automatically excluded and the mean of the four middle readings are displayed as the final result by the device. For each animal and IOP monitoring session the mean of two consecutive measurements was recorded if they were within 2 mmHg of each other; if there was more than a 2 mmHg difference, then the median of three measurements was recorded.

### Ocular coherence tomography (OCT)

Imaging and quantification of the RNFL thickness was performed using spectral-domain OCT; Heidelberg Eye Explorer 1.10.4.0 (Heidelberg Engineering GmbH, Heidelberg, Germany). This device combines confocal scanning laser ophthalmoscopy and conventional OCT technology (Spectralis Viewing Module Calculator 1.0.16.0, HRA2/Spectralis Family Acquisition Module 6.12.4.0, HSF Region Finder Module 2.6.4.0). The study animal was anesthetized using Ketamine-Xylazine cocktail and placed on a stage for imaging. Anterior chamber angle imaging was performed using the Spectralis anterior segment module. After anterior segment imaging, one drop of 1% Tropicamide (1% Mydriacyl, Alcon, Fort Worth, TX, USA) was instilled for mydriasis to obtain high quality retinal images. A laser beam with a wavelength of 870 nm is emitted by a super luminescence diode. The machine can acquire up to 40,000 A-scans per second with a transversal resolution of 14 μm and a depth resolution of 7 μm. The quality of the images is enhanced by an eye-tracking system (TrueTrack™, Heidelberg Engineering, Heidelberg, Germany) and automatic real-time averaging mode (ART). A 3.5 mm circle scan around the optic disc was used to acquire the circumpapillary RNFL thickness. To ensure that high quality images alone were analyzed, images with quality metric of 25 or more alone were considered. Segmentation lines at the internal limiting membrane and posterior border of RNFL were verified to ensure correct segmentation. The outer retinal thickness was defined as the thickness of retinal tissue between the external limiting membrane and the Bruch’s membrane.

### Optomotry

Assessment of visual function in the study animals was performed using an Optomotry system version 1.8.0 (CerebralMechanics, Lethbride, Alberta, Canada); a non-invasive device for assessing the optokinetic response of the animal in response to dynamic vertical sine wave gratings projected on the four walls of a cuboidal enclosure. The animal is placed on an elevated stage and the visual function is measured based on the optokinetic movement of the head in response to the moving patterns, which is observed via an overhead camera. Since rats track movement from temporal to nasal fields alone, visual function of each eye can be assessed^34,35^. As both eyes of the same study animal were always either responders or poor responders, mean visual acuity of both eyes (combined visual acuity) was considered for analyses. Vision was assessed at baseline before suturing and 17 weeks.

### Pathological analyses and imaging

After euthanasia, eyes were fixed in 4% paraformaldehyde, equilibrated in 30% sucrose, embedded in optimal cutting temperature compound and cryosectioned. 12-μm sections were blocked with 5% donkey serum and probed with primary antibodies to RBPMS (PhosphoSolutions, Catalog # 1832-RBPMS), GFAP (Sigma-Aldrich, G3893), MMP-2 (Proteintech, Catalog #10373-2-AP), MMP-9 (Abcam, ab38898), CD68 (BioLegend, Catalog #137001), CD31 (Abcam, ab28364), F4/80 (BioLegend, Catalog #122601), CD3e (Invitrogen, MA1-90582) and CD4 (Invitrogen, 14-0040-82) according to standard protocols. The sections were washed with PBS-Tween and incubated with fluorescent-conjugated secondary antibodies (Molecular Probes) and DAPI. Subsequently, sections were mounted using MOWIOL 4-88 (Millipore Sigma). Immunofluorescent images were acquired with a Nikon Eclipse-Ti confocal microscope, and analyzed with NIS Elements software version 4.51. RGC survival analyses was performed on cryosections to maximize opportunity for additional histological studies, as we have previously described^20,27^. Briefly, retinal sections at the level of the optic nerve were imaged and RBPMS-positive cells were counted along 250 μm on both sides of the optic nerve insertion. At least five images each from different sections on both sides of the optic nerve insertion were analyzed for each eye and counts were averaged to RBPMS-positive cells per 100 μm/eye. For some experiments eye tissues (angle tissues, retina, and optic nerve as indicated), were collected and homogenized in aliquoted microfuge tubes, and then snap frozen at − 80 °C. Samples were then submitted to quantitative multiplex laser bead analyses (Bio-Plex 200) for assessment of a 27-plex rat cytokine panel and a 3-plex TGF-β panel (Eve Technologies).

### Statistics

For all experiments, *n* refers to the number of eyes or biological replicates. Graphpad Prism 8.4.3 was used to generate graphs. RGC survival analysis, IOP trend comparisons and visual acuity comparisons between different groups were performed using the unpaired t-test. RNFL thickness, outer retinal thickness and visual acuity comparisons between the same group were performed using the paired t-test. Cytokine analyses was performed using one-way ANOVA with Tukey’s post-hoc analyses. A p-value of less than 0.05 was considered statistically significant.

## Results

### The snug suturing technique results in gradual and sustained ocular hypertension

**Figure 1:**
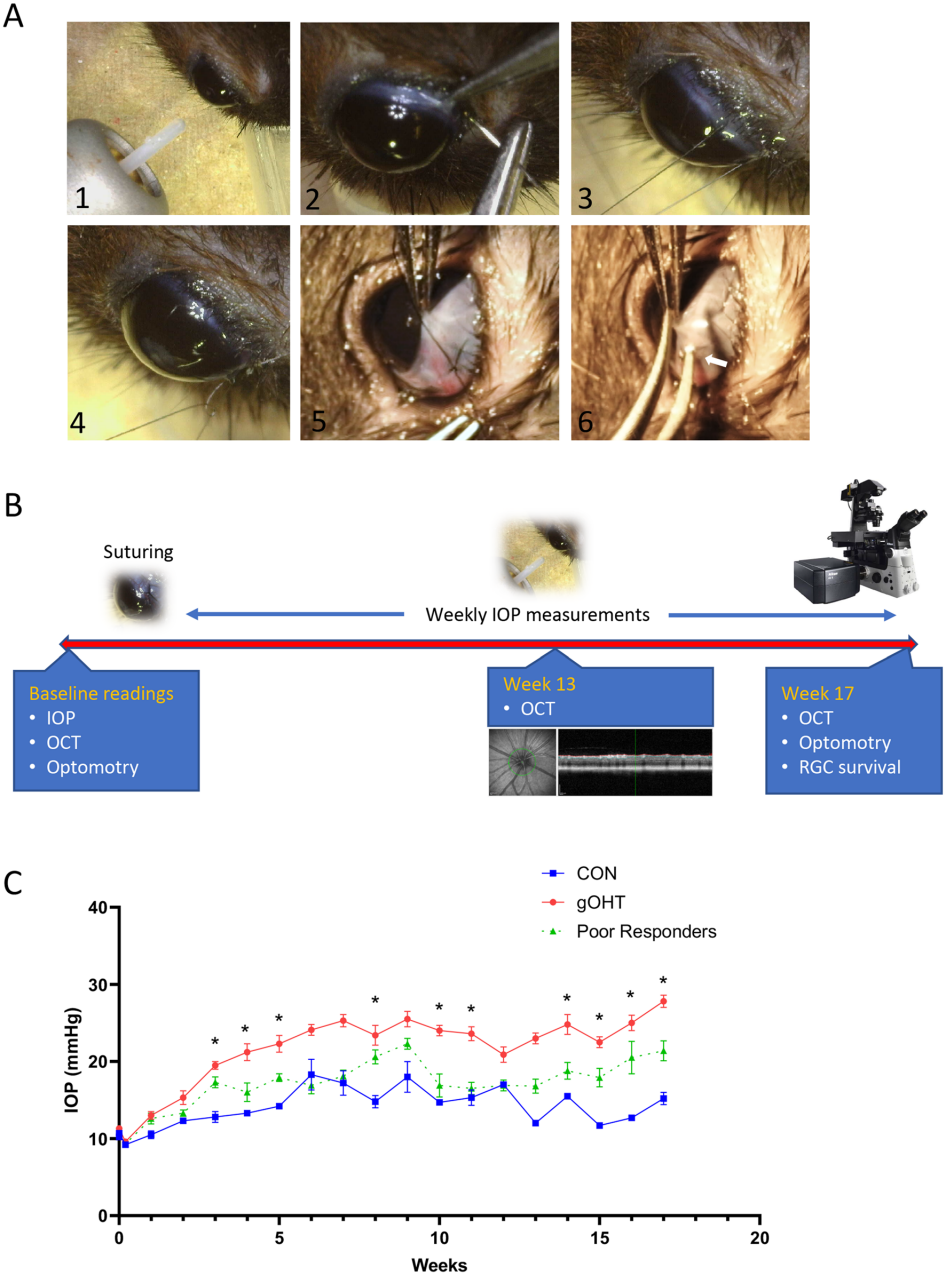
The snug suturing technique results in gradual and sustained ocular hypertension. (**A**) Representative pictures of the procedure; IOP is measured by rebound tonometer (1), nylon 8–0 suture was passed subconjunctivally (2, 3) and tied using a slip-knot (4) to achieve a snug fit (5). One arm of a forceps could be easily passed under the suture (white arrow), demonstrating a snug fit (6) (pictures taken with a Dino-Lite Edge Polarizing Digital Microscope). (**B**) Timeline for the associated study indicating baseline and follow-up measurements. (**C**) Average IOP curves showing significant elevation in the gOHT group compared to CON (n = 12 and 8 eyes for gOHT and CON, respectively, *p < 0.001, bars are S.E.). CON; control, gOHT; gradual ocular hypertension, IOP; intraocular pressure, OCT; optical coherence tomography, RGC; retinal ganglion cell.

Eyes of 6-week-old Long Evans rats were sutured around the limbus and carefully tightened to fit ‘snugly’, without an immediate effect on IOP, as detailed in the methods (Fig. 1A). Control animals (CON) also underwent surgery, but the sutures were left lax. Once the procedure was refined, we designed an experiment in which snug-sutured and control eyes were followed up with weekly IOP measurement. Longitudinal OCT and optokinetic measurements were taken over 17 weeks, followed by histological analyses (Fig. 1B). Notably, at two minutes post-suturing, the IOP’s did not change significantly from baseline for either controls (10.2 ± 0.4 to 9.2 ± 1.4 mmHg) or snugly-sutured gOHT eyes (10.5 ± 1.0 to 9.6 ± 0.7 mmHg) (Supplementary Fig. 1). Following suturing, ocular hypertensive eyes were defined as having at least 80% of IOP measurements from 5 weeks post-suturing more than or equal to 20 mmHg. Snug-sutured rat eyes maintained near-baseline IOPs up to 2 weeks post-suturing. The IOPs at weeks 1 and 2 were 13.0 ± 1.7 and 15.3 ± 3.0 mmHg, respectively. Starting at 2 weeks following surgery responder eyes developed a gradual increase in IOP over time (Fig. 1C). The IOPs showed a significant increase as early as 3 weeks post-suturing, and by 5 weeks, a consistent IOP elevation of 20 mmHg or higher was reached and was maintained for an additional 12 weeks (adding to 17 weeks total). Of 20 snug-sutured eyes, 12 met our inclusion criteria and demonstrated consistent elevated IOP. Eight eyes that did not show a consistent IOP response were excluded from the gOHT group as non-responders. In comparison, loosely sutured control eyes generally exhibited consistent IOP measurements below 20 mmHg. Of eight loosely sutured eyes, six were observed to have at least 80% of IOP measurements below 20 mmHg. However, two loosely sutured eyes subsequently had elevated IOPs like the snug-sutures, with values that gradually rose above 20 mmHg and were excluded. These results indicate that the gOHT model induces gradual and consistent elevated IOP until 17 weeks in responder eyes (60% of the total). Interestingly, both eyes of the same study animal were always either responders or poor responders. A mixed outcome was not observed for any animal.

### The gOHT model results in progressive RNFL thinning and decreased vision after 17 weeks

**Figure 2:**
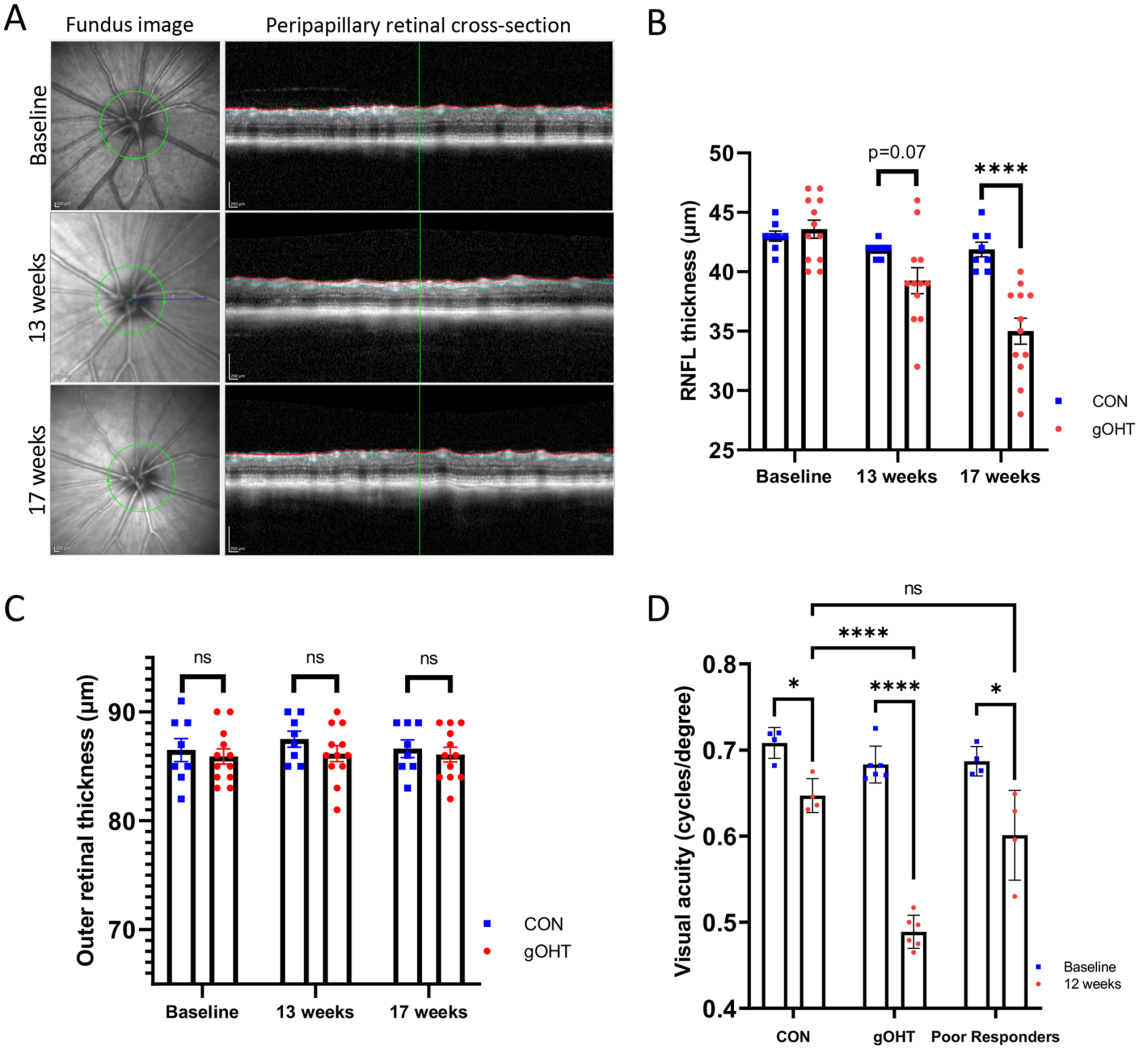
The gOHT model results in progressive RNFL thinning and decreased vision. (**A**) Representative fundus and corresponding cross-sectional OCT images used to measure the RNFL thickness at baseline, and at 13 and 17 weeks after surgery (scale bars represent 200 μm). The green circle represents the cross-sectional circumference and red and blue tracings outline the RNFL. (**B**) Quantified RNFL thickness measurements indicate progressive thinning in the gOHT group compared to CON that became highly significant after 17 weeks. ‘Snugly’ sutured eyes that showed a poor IOP response were excluded from the gOHT group (n = 8 and 12 for CON and gOHT, respectively, ****p < 0.0001, bars are SE). (**C**) Outer retinal thickness measured using OCT did not show statistically significant change at any time point (ns; not significant, bars are SE). (**D**) Comparison of visual acuity after 17 weeks (12 weeks of elevated IOP) in CON and gOHT groups shows a highly significant difference. However, there was a statistically significant decrease in vision in all groups post-suturing (p < 0.05). Notably, animals that responded poorly in terms of IOP elevation post-suturing did not show a statistically significant difference from CON (n = 4, 6 and 4 for CON, gOHT, and poor responders, respectively, ****p < 0.0001, *p < 0.05, bars are SE). RNFL, retinal nerve fiber layer.

Prolonged elevation of IOP in clinical glaucoma results in permanent inner retinal damage and corresponding visual field loss^36^. Other rodent models of glaucoma that exhibit prolonged elevated IOP also demonstrate moderate inner retinal damage, including the established tightened ocular hypertensive circumlimbal suture model^18,37^. Longitudinal retinal nerve fiber layer thickness (RNFL) was evaluated in our study using optical coherence tomography (Fig. 2A,B). On quantification, no change was observed between groups at baseline. Decreased RNFL thickness was observed in gOHT compared to CON animals after 13 weeks, but the difference was not statistically significant (p = 0.07). By 17 weeks, the RNFL showed progressive thinning, with a highly significant difference between CON and gOHT groups (p = 0.0002). In comparison, outer retinal thickness is not typically affected in glaucoma^38^. Therefore, to assess the specificity of damage, outer retinal thickness was measured to rule out a broad pan-retinal injury. The outer retinal thickness did not differ significantly between CON and gOHT at any time point (Fig. 2C).

As another functional measure of retinal damage, visual acuity was assessed longitudinally in CON and gOHT animals using optomotry. All groups experienced reduced acuity compared to baseline, presumably due to the suturing procedure and secondary ocular surface changes. However, the difference was more pronounced in the gOHT group (p < 0.0001) than the CON (p = 0.005) and poor responders groups (p = 0.02). Additionally, the gOHT group displayed a highly significant decrease in visual acuity compared to CON animals after 17 weeks (p < 0.0001, Fig. 2D). As an added comparison, we assessed the poor responders in the gOHT group that did not meet elevated IOP criteria. There was no significant difference in visual acuity between CON and poor responders after 17 weeks (p = 0.15), and these animals’ optomotry scores fell in between the two main groups. These data further implicate the role of elevated IOP in inner retinal injury-induced vision loss.

### Chronic gOHT results in inner retinal neuropathology

**Figure 3:**
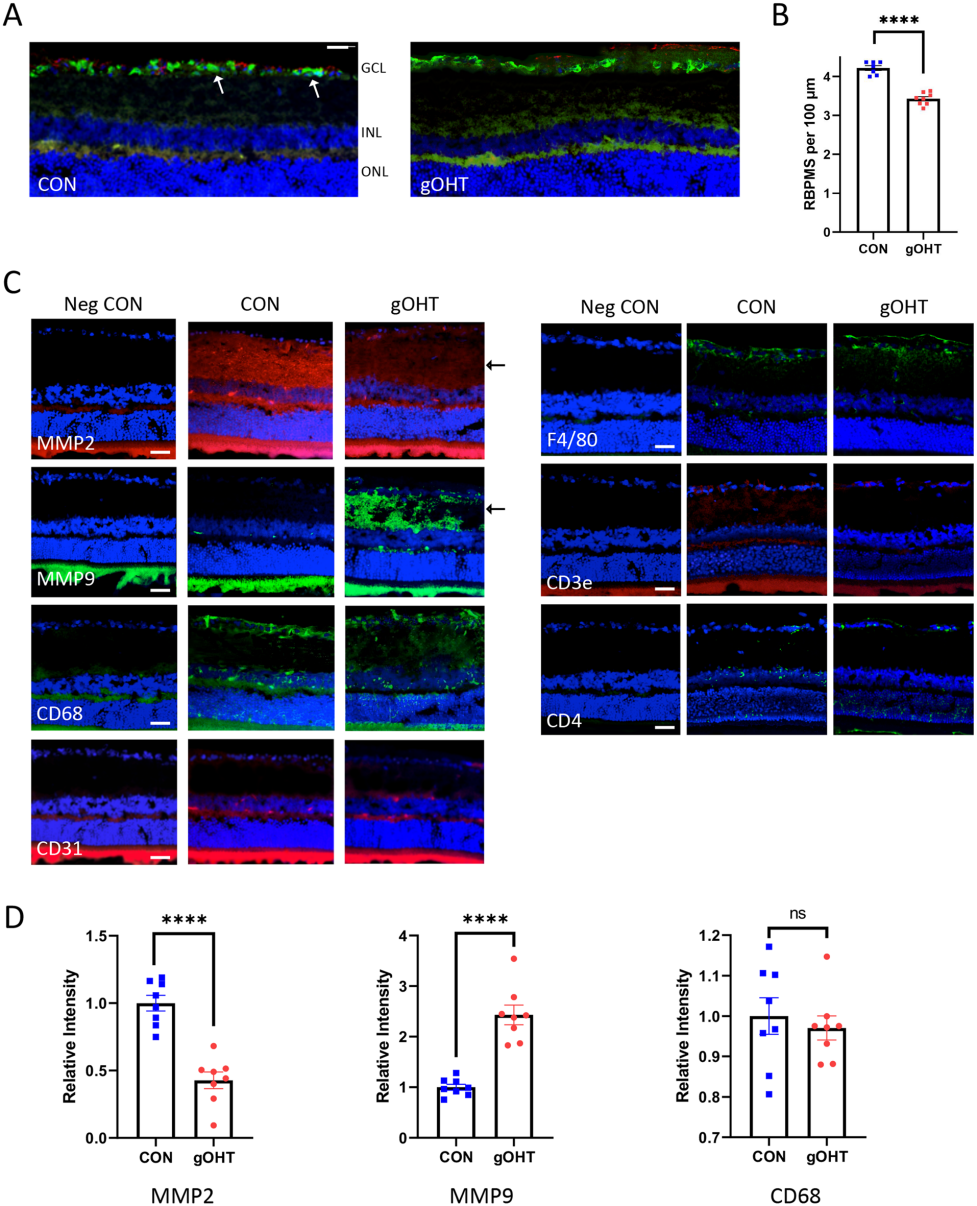
Chronic gOHT results in inner retinal neuropathology. (**A**) Representative retinal images from CON and gOHT eyes after 17 weeks in the gOHT group, stained for RBPMS (green, arrows) and GFAP (red), in addition to the nuclear marker DAPI (blue) (scale bar indicates 50 μm). (**B**) Corresponding quantification of RGC density indicates significantly decreased survival in gOHT compared to CON (****p < 0.0001, bars are SE). (**C**) Representative immunostaining panels of CON and gOHT retinas in the same orientation. MMP-2 signal decreased and MMP-9 signal is increased in the gOHT inner retina compared to CON eyes (arrows). There was no notable difference in staining for CD68, CD31, F4/80, CD3e or CD4 between gOHT and CON. Note: negative controls (Neg CON) are sections stained with a secondary antibody alone. (scale bars indicate 50 μm). (**D**) Quantification of staining across image sets shows a significant decrease for MMP2, a significant increase for MMP9, and no change for CD68 markers (n = 8, bars are SE, ****p < 0.0001, ns; not significant). GCL, ganglion cell layer; INL, inner nuclear layer; Neg CON, Negative Control; ONL, outer nuclear layer.

The inner retinal damage observed on OCT was confirmed after 17 weeks using histopathological analyses followed by confocal microscopy. Eyes were sectioned in order to maximize the options for analyses and RGCs were counted and averaged across multiple central sections for each eye after staining for the marker RBPMS (Fig. 3A). A moderate, but statistically significant 18.8% decrease in RGC survival was observed after 17 weeks in the gOHT group (p < 0.0001, Fig. 3B).

The inner retinal damage and related mechanism was further characterized by probing with antibodies raised against markers that would indicate wound healing or inflammatory processes. Markers were assessed for extracellular matrix remodeling (MMP-2, MMP-9), microglia (CD68), vascular endothelium (CD31), macrophages (F4/80) and CD3e and CD4 T-cells (Fig. 3C). Interestingly, a decrease in MMP-2 and an increase in MMP-9 was consistently observed in the gOHT retina compared to CON (p < 0.0001 for both), indicative of active tissue remodeling (Fig. 3D). Notably, no changes were observed for any inflammatory cell markers or microglia (CD68) in the gOHT retina compared to CON, which suggests that immune cell infiltrates are unlikely to be a primary driver of inner retinal injury (Fig. 3C,D). In particular, there was no evidence of T-cell invasion of the gOHT retina, which were assessed due to a recent report from another animal model^39^. Negative controls were stained using secondary antibody alone (Fig. 3C), and positive controls for each antibody were assessed on ocular tissues from a different ocular inflammation model (Supplementary Fig. 2). (Note, bright staining of photoreceptor outer segments on some sections are a secondary antibody artifact.) Together, these findings provide further support that chronic gOHT results in inner retinal injury and remodeling, with no evidence of inflammatory infiltrates.

### Chronic gOHT results in optic nerve head tissue remodeling and neuroinflammation

**Figure 4:**
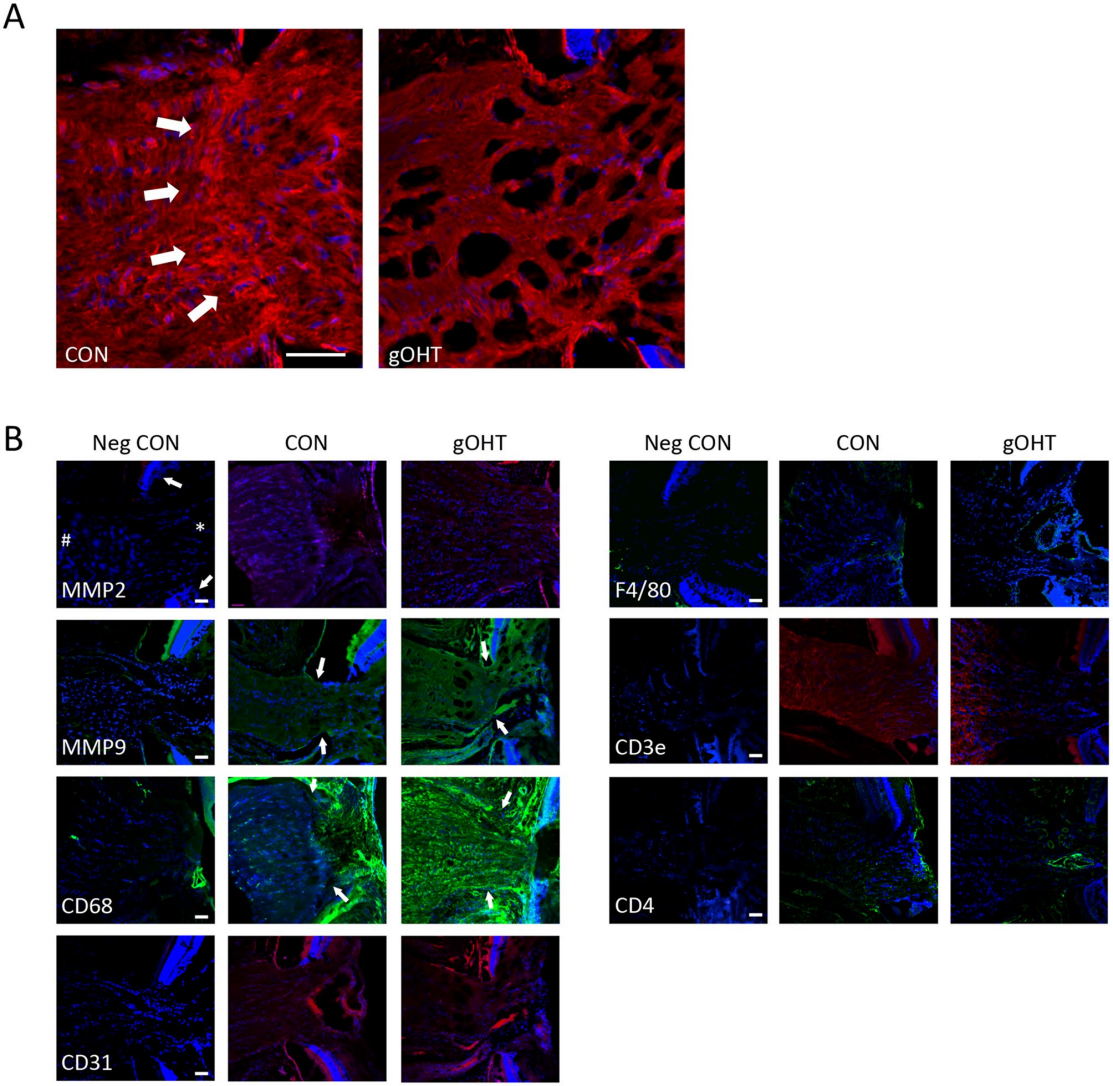
Chronic gOHT results in optic nerve head tissue remodeling and neuroinflammation. (**A**) Representative phalloidin staining (red) highlights an intact pseudolaminar region in control (CON) tissue (arrows), which was disrupted in gOHT eyes (scale bar indicates 50 μm). (**B**) Representative immunostaining panels of CON and gOHT in sections of optic nerve head tissue. For orientation of all images, the white arrow indicates DAPI stained retinal layers on either side, * indicates the vitreous cavity adjacent to optic nerve head, and # indicates the optic nerve). There was mildly increased staining for MMP-9, and strongly increased CD68 in gOHT compared to CON tissues. There were no notable differences for any other marker (scale bar indicates 50 μm).

The optic nerve head (ONH) was also analyzed by immunohistology and confocal microscopy. ONH pathology was evaluated using fluorescent-tagged phalloidin, which is a bicyclic heptapeptide that binds with high affinity to actin. An intact pseudolamina was clearly visible in CON optic nerve sections. However, in gOHT optic nerves, the organization of the pseudolamina was consistently disrupted, consistent with either damage in situ, or increased vulnerability during processing (Fig. 4A). Sections from gOHT group eyes showed a marked increase in CD68 staining in the pseudolaminar region compared to the CON group, suggestive of microglial activation (Fig. 4B). There was also an accompanying consistent increase in MMP-9 staining throughout the ONH, indicative of some tissue remodeling in response to chronic elevated IOP (Fig. 4B). These results are consistent with increased tissue remodeling, microglial activation, and disruption of the pseudolamina in the gOHT optic nerve.

### Eye growth does not contribute to increased IOP in the gOHT model

**Figure 5:**
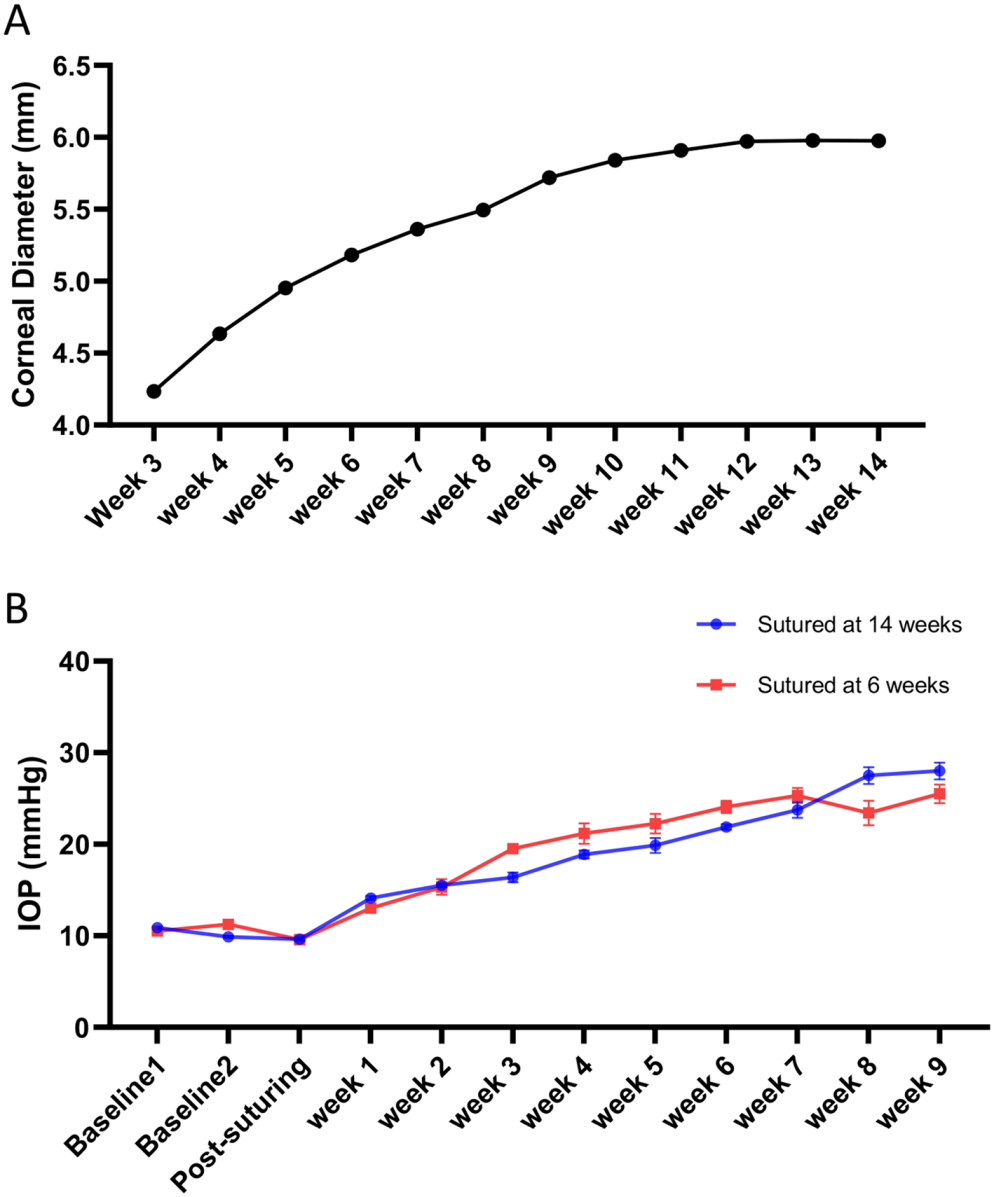
Eye growth does not contribute to increased IOP in the gOHT model. (**A**) Average corneal diameter was measured from 3 weeks of age until it plateaued at 12–14 weeks. Note the range of measured values were too narrow to display error bars (n = 8). (**B**) IOP measurements from eyes sutured at 14 weeks (n = 8) show the same increased IOP trend as eyes sutured at 6 weeks (n = 12, bars are SE).

We were curious as to the mechanism underlying the gradual suture tightening over time and evaluated eye growth as a potential explanation. As we could not find any studies in the literature on the corneal diameter increase with age in Long Evans rats, we measured this parameter weekly, starting at 3 weeks of age (n = 8). The measurements reached a plateau at 12–14 weeks at an average of 5.98 mm (Fig. 5A). Eyes were then sutured at 14 weeks and the IOP measurements were monitored weekly. We hypothesized that if eye growth were the primary cause of suture tightening, there would be no increase in IOP after growth had plateaued. However, we observed that the IOP increased in 14-week-old animals in a pattern similar to eyes sutured in 6-week-old animals (Fig. 5B). These results indicate that eye growth is not the primary etiology behind increased IOP in the gOHT model.

### There is no change in angle appearance or pathology between CON and gOHT eyes

**Figure 6:**
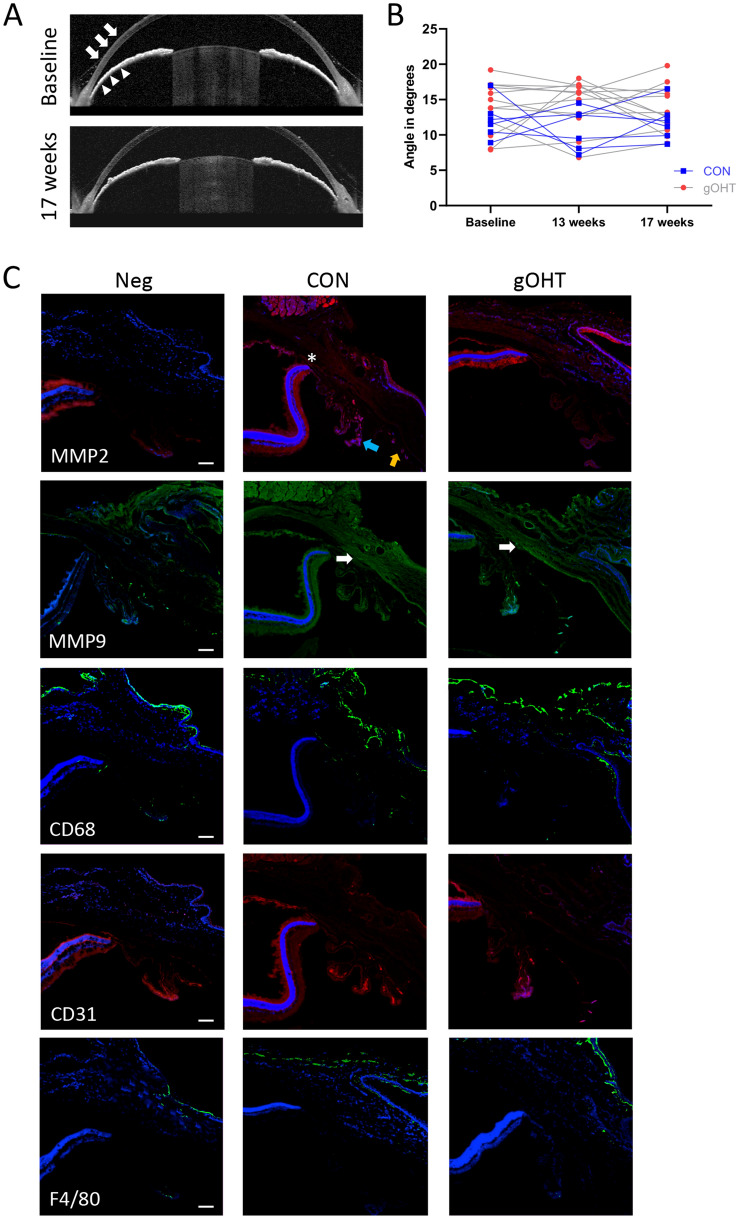
There is no notable change in angle appearance or pathology between CON and gOHT eyes. (**A**) Representative OCT images of the anterior chamber angle show the same eye at the area between the peripheral cornea (arrows) and iris (arrowheads). The angle remained open until the last follow-up after 17 weeks post-suturing. (**B**) Line graphs indicate that the anterior chamber angle did not significantly change over time in either CON or gOHT groups. (**C**) A panel of relevant immunofluorescent markers indicates no significant differences in staining between CON and gOHT angles for MMP-2, MMP-9, CD68, CD31 or F4/80 staining (scale bars indicate 50 μm, all images are shown in the same orientation; the white asterisk indicates the ora serrata, blue arrow points to the ciliary processes, orange arrow to the iris and white arrows to Schlemm’s canal.

Glaucoma is broadly classified into open angle or angle closure presentations^40^; the anterior chamber angle being subtended between the peripheral cornea and the iris. We used anterior chamber OCT to evaluate angle status in sutured eyes at baseline and after 17 weeks (Fig. 6A). All angle measurements were obtained in the nasal-temporal meridian. The angle measurements did not change significantly at any time point (Fig. 6B). The angle remained structurally open even at 17 weeks, indicating that this was an open angle glaucoma model.

Histologically, we did not observe any significant differences in MMP-2 and MMP-9 staining between gOHT and CON angles, or outflow tissues, indicating no significant difference in tissue remodeling (Fig. 6C). Similarly, there were no marked staining differences following probes for any inflammatory cell or vascular markers assessed, including CD68, CD31 and F4/80 (Fig. 6B).

### Retinal neuroinflammatory signaling differs in acute and gradual ocular hypertension models

**Figure 7:**
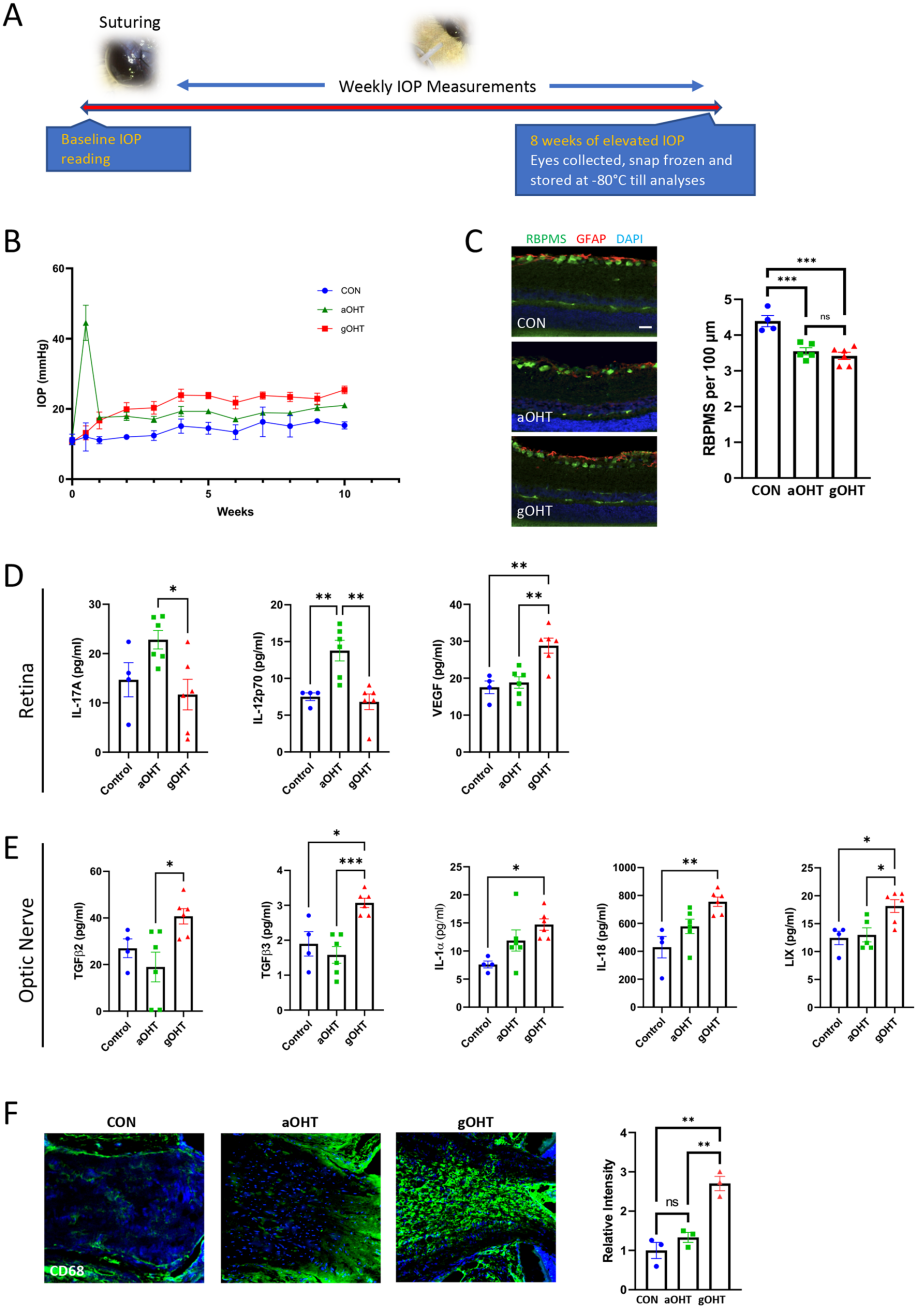
Retinal neuroinflammatory signaling differs in acute and gradual ocular hypertension models. (**A**) Experiment timeline showing weekly IOP measurements after suturing (CON, aOHT and gOHT groups), as well as pathology and cytokine profiling endpoints after 10 weeks post-suturing (pictures taken with a Dino-Lite Edge Polarizing Digital Microscope). (**B**) Recorded IOP curves show minimal change in IOP for the control (CON) group, for the aOHT group there was a marked transient spike in IOP at induction followed by tapering values, and for the gOHT group there was a gradual increase in IOP that remained consistently elevated above 20 mmHg from 3 weeks after suturing (n = 4, 6 and 6 for CON, aOHT and gOHT, respectively; bars are SE). (**C**) Representative retinal staining and RGC survival analysis shows significant and comparable loss in the aOHT and gOHT groups compared to CON (scale bar indicates 50 μm, ***p < 0.001, bars are SE, ns; not significant). (**D**) Multiplex cytokine analysis of retinal tissues revealed significantly elevated IL-17A in the aOHT group compared to gOHT. IL-12p70 levels were significantly elevated in the aOHT group compared to control and gOHT. In contrast, VEGF was significantly elevated in the gOHT group compared to control and aOHT. (*p < 0.05, **p < 0.01, bars are SE). (**E**) Multiplex cytokine analysis of the optic nerve identified significant elevation of TGF-β2, TGF-β3, IL-1α, IL-18 and LIX in the gOHT group, but no changes in the aOHT group (*p < 0.05, **p < 0.01, ***p < 0.001, bars are SE). (**F**) CD68 staining was significantly higher in the gOHT group compared to CON and aOHT groups (**p < 0.01, bars are SE). aOHT, acute ocular hypertension; IL, interleukin; LIX, TGF, transforming growth factor; TNF, tumor necrosis factor; VEGF, vascular endothelial growth factor.

After ruling out the roles of eye growth, or major changes to the angle, we set out to further characterize potential inflammatory signaling in gOHT eyes. An ocular hypertension model that uses a circumlimbal suture to generate an acute increase in IOP (aOHT) has previously been well-characterized^18,32^, presenting a pattern similar to most other inducible sustained IOP models^5,17,41^. Due to the close similarity in suturing techniques, but dramatic differences in resulting IOP trend after induction, we designed an experiment to compare the acute and gradual IOP models (Fig. 7A). Immediately following surgery the aOHT eyes displayed a sharp increase in IOP in comparison to gOHT eyes, which remained at baseline (Supplementary Fig. 1). In addition to gOHT and aOHT groups, we also included a CON group of loosely sutured eyes, which did not experience a significant increase in IOP. After suturing, weekly IOP measurements were recorded, and eyes were collected for analyses after 10 weeks post-suturing, representing a timepoint near the initiation of permanent retinal damage^18,20,32^.

As expected, the IOP trend differed markedly for the three groups: in the aOHT group there was an initial spike in IOP to 44.5 ± 5.0 mmHg after suturing, the gOHT group showed a gradual increase in IOP to over 20 mmHg 3 weeks post-suturing, and the CON group did not exhibit an increase in IOP (Fig. 7B). Secondary to the elevated IOP, there was a similar significant decrease in RGC survival for the aOHT and gOHT groups (p = 0.001 and 0.0002, respectively) compared to the CON group (Fig. 7C).

Multiple eye tissues were also collected from rats in each group for multiplex cytokine analysis to identify the neuroinflammatory signaling profiles for the three groups. From each eye, samples were collected from the angle, retina, and optic nerve, and each tissue was tested quantitatively for concentrations of 30 cytokines and chemokines using a multiplex immunoassay. Remarkably, although values were detected for most cytokines in each tissue sample, the majority of analytes showed no significant differences between groups, consistent with the absence of any major inflammatory response (Supplementary Table 1). Notably, in angle tissues there were no significant differences at all, suggesting that no suture-dependent differences in inflammation underlie the resulting pathology in OHT eyes.

However, those analytes that showed a statistically significant difference between groups are shown in Fig. 7D–F and revealed interesting patterns in retinal and optic nerve cytokine signaling. In particular, some differences were detected between aOHT and gOHT responses. In retinal tissues IL-17A, a pro-inflammatory cytokine closely associated with T helper cell function, was significantly elevated in only the aOHT group, compared to the gOHT group (p = 0.03). Similarly, IL-12p70, indicating the active heterodimer of a pro-inflammatory mediator involved in T helper cell differentiation, was significantly elevated in the aOHT group, compared to CON and gOHT (p = 0.009 and 0.002, respectively). These changes are notable due to recent prominent work implicating T helper cell activities in other glaucoma models^39,42^. In contrast, VEGF was the only analyte significantly elevated in retinal tissue from the gOHT group compared to CON and aOHT (p = 0.003 for both) suggesting a change in vascular permeability or growth.

Optic nerve tissue analyses also identified some interesting patterns which only identified significant changes in the gOHT group. There was a significant elevation in levels of TGF-β2 in the gOHT group compared to aOHT (p = 0.02), and TGF-β3 levels were also significantly elevated in the gOHT group compared to CON and aOHT (p = 0.01 and 0.0009, respectively). These factors are indicative of tissue injury and repair responses and their elevation is consistent with elevated TGF-β2 and TGF-β3 levels in primary open angle glaucoma (POAG) patients compared to controls^43^. In addition, IL-1α and IL-18, which are both broad pro-inflammatory cytokines from the IL-1 family that has also been previously linked to POAG^44,45^, were elevated in the gOHT group compared to CON (p = 0.01 and 0.003, respectively). LIX (also known as CXCL5), is a chemokine associated with tissue remodeling that amplifies a rapid stress response via a phosphatidylinositol 3-kinase-NF-kappa B pathway^46^, and was similarly elevated in the gOHT group compared to CON and aOHT (p = 0.02 for both). As these changes may be linked to the prominent microglial response we had observed earlier in the ONH (Fig. 4B), we also compared this signal in the two models. Analyses of CD68 staining in ONH tissues from each groups subsequently uncovered a significant increase in gOHT eyes compared to aOHT and control eyes (p < 0.01, Fig. 7F). Therefore, although the cytokine analyses broadly confirmed the absence of a robust proinflammatory response in sutured eyes, it also revealed surprising differences in retinal and optic nerve injury signaling between the aOHT and gOHT models.

## Discussion

Limitations among common inducible rodent glaucoma models include a potentially pathological spike in IOP at induction, associated inflammatory response, and short duration of elevated IOP requiring multiple interventions^5^. In comparison, the gOHT suture model presented many features that more closely recapitulate clinical glaucoma. For example, it resulted in gradual and sustained ocular hypertension, with IOPs consistently above 20 mmHg for at least 17 weeks post-suturing without the need for multiple interventions. Subsequent pathological analyses showed no evidence of retinal inflammatory cell infiltration. This stress resulted in moderate but significantly decreased RGC survival, selective inner retinal injury and decreased visual function. Eye growth, anterior chamber angle closure, or inflammation in the tissues around the anterior chamber angle, were each ruled out as etiologies for suture tightening over time and resulting elevation in IOP. Within the optic nerve, there was disruption of the pseudolamina, increased microglial activation and evidence of tissue remodeling through actin reorganization and supported by increased TGF-β2 and TGF-β3 levels and increased MMP-9 expression. Neuroinflammation involving the optic nerve was further supported by increased levels of proinflammatory cytokines IL-1α and IL-18.

A variety of animal models that feature increased IOP and relevant pathology can be broadly classified into either genetic or inducible approaches. Genetic models often feature mutations that affect the normal functioning of the RGCs or aqueous humor dynamics^6^. Thus, genetic models may be ocular hypertensive, such as the DBA/2J mice^47^ and CTGF overexpressing transgenic mice^48^, or normotensive, such as mice deficient in glutamate transporters GLAST or EAAC1^49^. Yet, many of these models are very slow in onset and feature variability in phenotype and pathology. Importantly, it can also be unclear whether the underlying mutation alters the biology of the eye such that clinical relevance is diminished. In contrast, inducible models offer a rapid and consistent onset of increased IOP in wild type animals, but have limitations, such as inflammation, often a severe IOP spike at induction and need for multiple interventions to maintain elevated IOP.

One characteristic advantage of the gOHT model is the absence of a high spike in IOP at induction, which is a common feature of nearly all other existing inducible models. Reported spikes after induction range from 25 to 76 mmHg^5,7,10,19^, followed by gradual or rapid return to baseline. In contrast, the gOHT suture model exhibits a gradual increase in IOP over time, similar to most eyes with untreated clinical glaucoma. Methods have been developed to generated sustained IOP increases in other inducible models. For example, in the microbead model, Chen et al. demonstrated that the duration of IOP elevation could be prolonged from 4 to 8 weeks by an additional intracameral injection of microbeads^50^. Similarly, laser photocoagulation of the trabecular meshwork and intracameral viscoelastic injection have been repeated multiple times after induction to maintain elevated IOP for a longer duration^5^. However, potential complications of repeated laser application or intraocular procedures include anterior chamber flattening, formation of corneal neovascularization and opacification, inflammation and permanent mydriasis^6,51^. A single intervention at induction without the need for supplementation also minimizes the influence of confounding factors, such as inflammation. Many rodent models, such as the microbead model, hyaluronic acid injection, and anterior chamber cannulation, require intraocular entry, creating opportunity for inflammation and blood-aqueous barrier compromise^5^. Procedures that do not involve intraocular entry, such as episcleral vein cauterization and laser photocoagulation can also result in significant inflammation^5,6,51,52^. In comparison, the circumlimbal suturing technique involves minimal conjunctival compromise with no intraocular entry, thus reducing the risk of inflammation. Indeed, examination of the angle tissues for cell infiltrates or inflammatory cytokines did not reveal any significant difference between the gOHT eyes and loosely sutured controls.

Glaucoma models can be broadly classified into those driven by an IOP-dependent mechanism, such as all of the models mentioned so far, and those driven by IOP-independent mechanisms, such as optic nerve crush^21,22,53^, excitotoxic retinopathy model^27,54,55^ and the ischemia–reperfusion model^30,56,57^. The gOHT model is an IOP-driven model and our data largely ruled out eye growth, angle closure and a generalized inflammatory response as etiologies for elevated IOP. Wong et al. demonstrated that tight circumlimbal suturing increased the episcleral venous pressure by compression of the episcleral vascular network, resulting in aqueous outflow resistance and elevated IOP^58^. A similar finding of elevated episcleral venous pressure was reported in POAG (12.1 ± 0.5 mmHg) and normal tension glaucoma (11.6 ± 0.4 mmHg) patients compared to controls (9.5 ± 0.2 mmHg)^59^. Therefore, we surmise that a similar, but gradual increase in episcleral venous pressure likely underlies the gOHT model.

Interestingly, the histological and cytokine profiling signature was unique for the optic nerve compared to retinal tissues in the gOHT model. In the optic nerve, TGF-β2, TGF-β3, IL-1α, IL-18 and LIX were significantly elevated at 8 weeks, when RGC loss was starting. The TGF-β superfamily consists of multifunctional cytokines with important roles in the cell cycle, differentiation, apoptosis, immunoregulation and deposition and degradation of the extracellular matrix (ECM)^60,61^. In particular, TGF-β2 has been established to play a significant role in human optic nerve head ECM remodeling via the Smad signaling pathway^62^. Additionally, in vitro supplementation of TGF-β2 increased the synthesis of elastin, collagen and fibronectin in human ONH astrocytes^62–64^. However, TGF-β3 function has been far less well-studied in this context. Tissue remodeling in the gOHT optic nerve was also supported by increased MMP-9 staining. ONH astrocytes express MMPs 1, 2, 3, 7, 9, and 12, along with their inhibitors TIMP 1 and 2^64–67^, and glaucomatous changes in the optic nerve could be due to an imbalance in ECM production and degradation^68^. Increased levels of MMPs has been reported in post-mortem glaucomatous optic nerve heads^69^. However, MMPs are secreted as zymogens and therefore, increased synthesis may not necessarily translate to increased ECM degradation^68,70^.

IL-1α and IL-18 belong to the same IL-1 family and are predominantly proinflammatory in function. IL-1α may act early in inflammation via neutrophil recruitment^71^. Microglial release of IL-1α has been demonstrated after optic nerve injury and results in induction of neuroinflammatory reactive astrocytes^72,73^. Elevated levels of IL-18 have also been reported in inflammatory and autoimmune diseases^74^. In addition to promoting a cascade of cytokine release, IL-18 activates NF-κB to induce NO release, and has prodegradative actions^75–77^. Elevated IL-18 has been reported in the aqueous humor of DBA/2J mice^45^ and in axons following optic nerve crush injury^78,79^. Similarly, LIX (also known as CXCL5), is a chemokine associated with tissue remodeling and which amplifies a rapid stress response via a phosphatidylinositol 3-kinase—NF-κB pathway^46^. CXCL5 has also been reported to activate resident microglia and attract inflammatory cells and has been put forward as a potential therapeutic target for optic nerve injury^80,81^. Together with the observed increase in microglial response, these activities and cytokines are consistent with a parainflammatory response in the optic nerve and ONH driven by gradual ocular hypertension to induce a degenerative tissue remodeling and injury response process.

A further advantage of the suture methodology was the unique ability to compare the results of different IOP profiles directly for the first time. Thus, an acute IOP increase, similar to most inducible glaucoma models, was compared with a gradual IOP increase simply by controlling suture tightness. Remarkably, although the profiles of resulting RGC loss appeared similar in both approaches, the accompanying cytokine profiles revealed notable differences. For both model versions there were no significant changes in most cytokines, and no changes at all in the angle tissues, suggesting the absence of a major inflammatory response. However, in the aOHT retina, two cytokines linked to T-cell function, IL-17A and IL-12, were significantly elevated compared to gOHT and controls. IL-17A is the primary cytokine secreted by Th17 cells, a subset of CD4^+^ helper T cells^82^. In glaucoma patients, although IL-17A plasma levels were not significantly altered, elevated IL-17A-secreting cells were observed^83,84^. IL-12 is also a proinflammatory cytokine that regulates T-cell and natural killer cell responses, promotes Th1 differentiation and plays a role in linking adaptive immunity and innate resistance^85^. IL-12 levels have been reported to be elevated in aqueous humor of glaucomatous eyes^86,87^. Interestingly, glaucomatous neurodegeneration caused by infiltration of CD4^+^ T-cells primed by commensal microflora has been reported in a microbead mouse model, which generates an IOP curve similar to the aOHT model^39,42^.

In contrast, we did not observe increased IL-17A, IL-12, or T cell infiltration in the gOHT model retinas, which only showed increased levels of VEGF compared to controls. Increased VEGF levels may indicate evidence of retina vascular leakage, although no accompanying changes in CD31 staining or retinal swelling were evident. However, these data may be complex to interpret, as VEGF dependent activation of the PI3K/ACR pathway has also been alternately reported to both increase RGC survival^88,89^, and conversely to be a marker for it, with VEGF antagonism attenuating optic nerve injury^90^. Additionally, none of the optic nerve changes observed with gOHT were significant after aOHT. Therefore, these data remarkably suggest that aOHT and gOHT stresses may induce different pathological mechanisms. Clinically, acute angle closure attacks mimic the IOP pattern induced in aOHT, while primary angle closure glaucoma without acute attacks has an IOP trend similar to that induced in gOHT. Interestingly, the extent and pattern of optic nerve head damage were different in primary angle eyes with and without angle closure attacks^91^.

However, the gOHT model is not without limitations. Of 20 snugly sutured eyes, 12 had successful induction of chronic ocular hypertension, a success rate of 60%. The rate of induction may be further increased by refining consistent and meticulous suturing technique. Due to a lack of other inducible models that exhibit a gradual increase in IOP, our findings from the gOHT model, and their differences with aOHT, could not be easily compared across other methods with a similar profile. However, we note that the gradual and sustained IOP increase, open angles, modest progressive inner retinal pathology, optic nerve injury, and minimal inflammatory cell infiltration are all consistent with features of primary open angle glaucoma. It will be interesting to further apply this model to study optic nerve injury mechanisms due to gradual ocular hypertension, observe how the gOHT model responds to the use of established anti-glaucoma medications, and test potential neuroprotective strategies.

## Supplementary Information

Supplementary Information.

Supplementary Legends.

Supplementary Video S1.
