# Supplementary material for: An inducible rodent glaucoma model that exhibits gradual sustained increase in intraocular pressure with distinct inner retina and optic nerve inflammation
Source: Sci Rep. 2021 Nov 24;11:22880. doi: 10.1038/s41598-021-02057-w (PMC8613281; doi:10.1038/s41598-021-02057-w)
Supplement: Supplementary file 1 — Supplementary Information. [file 41598_2021_2057_MOESM1_ESM.pdf]

Supplementary Figures

An Inducible Rodent Glaucoma Model that Exhibits Gradual Sustained Increase in Intraocular Pressure with Distinct Inner Retina and Optic Nerve Inflammation

David J. Mathew, Izhar Livne-Bar, and Jeremy M Sivak

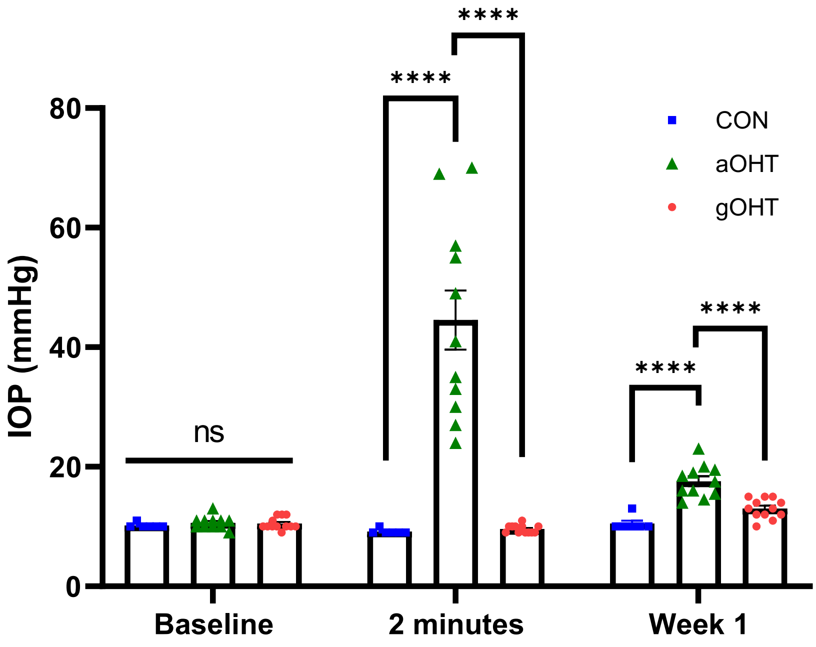

Supplementary Figure 1: Animals sutured with the tightened aOHT method result in induction of a strong IOP spike immediately following surgery, but snugly sutured gOHT eyes do not result in any change in IOP after one week (n>10, bars are S.E.).

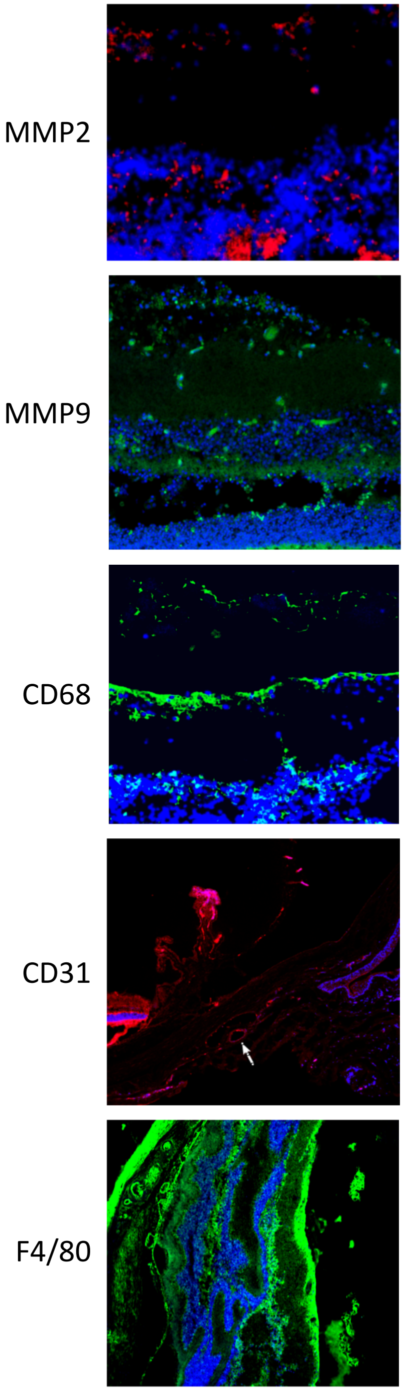

Supplementary Figure 2: Positive confocal microscopy controls generated using intravitreal paraquat injection to induce inflammation in rat eyes 48 hours prior to tissue processing.

	CON optic nerve		aOHT optic nerve		gOHT optic nerve		CON retina		aOHT retina		gOHT retina		CON angle tissue		aOHT angle tissue		gOHT angle tissue	
	Mean	SEM	Mean	SEM	Mean	SEM	Mean	SEM	Mean	SEM	Mean	SEM	Mean	SEM	Mean	SEM	Mean	SEM
**TGF β1**	8.65	1.44	5.84	1.99	11.64	2.15	7.70	0.95	5.73	0.95	5.72	1.58	32.42	11.01	45.36	9.29	57.75	5.42
**TGF β2**	26.99	4.02	18.95	6.37	**40.71**	**3.31**	235.20	74.05	121.20	39.28	190.57	64.35	82.20	29.88	113.47	18.85	116.49	18.78
**TGF β3**	1.90	0.35	1.58	0.24	**3.07**	**0.13**	3.32	0.37	2.67	0.24	3.08	0.55	6.14	2.12	8.52	1.34	10.00	1.07
**G-CSF**	-	-	80.59	16.78	64.65	9.06	70.12	6.31	98.23	11.72	99.04	10.21	41.75	0.00	117.12	11.00	91.90	10.88
**Eotaxin**	2.26	0.49	1.99	0.25	2.15	0.18	3.94	0.32	4.16	0.27	3.07	0.52	4.12	0.36	4.87	0.56	4.36	0.26
**GM-CSF**	17.34	3.20	11.42	2.17	13.47	2.02	21.79	5.55	23.37	1.60	15.59	3.67	3.56	1.59	11.75	3.06	4.46	2.69
**IL-1α**	7.60	0.62	11.87	1.88	**14.71**	**1.01**	18.52	4.20	22.79	2.87	21.72	3.26	15.76	0.76	20.95	2.25	35.78	9.50
**Leptin**	494.18	102.67	437.04	66.59	623.99	115.95	335.95	5.64	334.47	12.89	348.54	25.96	1054.30	163.05	1037.84	153.60	1158.90	112.72
**MIP-1α**	1.33	0.08	1.28	0.23	2.49	0.59	2.14	0.43	2.92	0.42	2.43	0.47	1.47	0.22	3.84	0.88	10.28	6.51
**IL-4**	2.81	0.00	5.85	1.21	2.81	0.00	7.54	2.47	11.53	1.18	7.84	1.44	7.51	2.36	9.21	1.37	6.59	1.32
**IL-1β**	34.36	5.57	29.08	3.85	40.42	7.28	84.85	6.35	98.04	3.40	94.25	5.10	97.64	7.75	99.76	6.95	197.04	60.06
**IL-2**	9.34	2.87	5.75	1.25	14.36	1.52	36.55	12.31	47.01	10.76	34.09	4.20	11.13	4.44	26.19	6.46	12.93	1.73
**IL-6**	-	-	-	-	73.72	0.00	329.54	66.02	583.33	161.21	545.96	161.87	73.72	0.00	146.58	36.43	303.25	101.12
**EGF**	1.81	0.42	2.50	0.52	3.57	1.14	1.21	0.53	0.55	0.13	1.17	0.18	3.54	0.45	6.08	2.38	8.52	2.44
**IL-13**	1.43	0.67	2.77	0.80	2.46	0.97	7.90	3.11	12.74	2.38	10.26	0.69	1.81	1.02	4.79	1.00	3.27	1.00
**IL-10**	11.85	2.54	12.28	1.22	16.05	0.94	22.03	2.98	32.53	1.93	28.87	2.79	13.63	2.17	23.69	2.72	19.38	1.66
**IL-12p70**	6.29	0.92	5.25	0.88	5.42	1.09	7.51	0.52	**13.77**	**1.39**	6.82	1.06	3.60	0.26	6.29	1.92	7.41	2.08
**IFNγ**	70.81	6.18	77.07	12.21	80.05	5.83	121.66	16.81	142.56	12.29	131.94	12.84	114.55	10.61	130.46	13.53	112.13	10.05
**IL-5**	13.89	2.57	11.14	1.05	9.96	1.58	32.99	5.25	43.80	3.23	28.51	5.24	17.30	2.48	15.83	2.55	17.40	2.86
**IL-17A**	1.19	0.41	0.18	0.00	1.18	0.69	14.70	3.47	**22.82**	**1.88**	11.69	3.11	2.25	0.13	2.51	0.49	4.11	1.04
**IL-18**	429.73	76.81	578.71	51.39	**755.57**	**33.58**	137.16	6.28	151.42	20.05	183.80	5.81	2042.14	172.44	2410.98	175.50	2240.96	270.88
**MCP-1**	40.77	11.47	25.47	5.50	67.53	4.83	120.29	44.28	128.30	26.22	91.72	11.40	59.89	6.24	66.25	14.29	49.69	8.30
**IP-10**	7.24	0.61	7.73	0.52	7.23	0.20	10.41	0.25	13.40	2.33	12.74	2.17	10.67	0.20	11.88	1.15	16.90	6.64
**GRO/KC**	130.85	22.91	109.92	9.13	112.71	2.11	140.38	13.45	167.50	13.31	148.07	11.39	126.04	14.95	129.32	16.19	103.33	10.44
**VEGF**	2.44	1.28	3.47	1.20	2.04	0.71	17.55	1.71	18.84	1.56	**28.82**	**2.02**	11.35	2.49	11.61	2.16	19.48	2.91
**Fractalkine**	10.17	1.36	12.39	1.48	16.91	2.32	50.90	4.85	52.53	7.99	56.76	6.06	95.64	9.62	94.42	10.04	111.38	18.21
**LIX**	12.44	1.21	12.99	1.26	**18.16**	**1.15**	18.38	4.32	24.55	2.86	20.04	3.36	16.43	2.06	27.25	3.66	41.68	9.20
**MIP-2**	30.53	6.01	29.79	3.04	31.95	6.13	46.29	7.47	44.15	4.95	38.02	5.63	9.76	0.00	-	-	100.46	82.11
**TNFα**	8.39	5.69	3.61	1.57	3.10	0.76	34.20	3.37	39.00	7.29	42.02	10.74	34.67	3.13	31.26	5.86	19.16	2.06
**RANTES**	1.09	0.19	1.00	0.13	0.90	0.17	1.02	0.09	0.96	0.06	0.89	0.13	8.14	0.82	7.30	1.25	9.24	2.46

Supplementary Table 1: Complete cytokine panel results for CON, aOHT and gOHT eyes. Values are in pg/ml, and those that are significantly different from control ($p<0.05$) are emboldened.
