# Supplementary material for: An inducible rodent glaucoma model that exhibits gradual sustained increase in intraocular pressure with distinct inner retina and optic nerve inflammation
Source: Sci Rep. 2021 Nov 24;11:22880. doi: 10.1038/s41598-021-02057-w (PMC8613281; doi:10.1038/s41598-021-02057-w)
Supplement: Supplementary file 2 — Supplementary Legends. [file 41598_2021_2057_MOESM2_ESM.docx]

**Supplemental Video 1**

**Circumlimbal suturing technique:** Baseline intraocular pressure (IOP) measurements are taken using a rebound tonometer. After anesthesia with intraperitoneal Ketamine-Xylazine cocktail, the rodent eye is sutured using Nylon 8-0. The needle is passed subconjunctivally 1.5 mm posterior to the limbus all around the eye, taking care not to penetrate the sclera. The suture is tied off gently using a slipknot without any tightening. This snug suturing technique ensures that there is no IOP spike immediately after suturing. One method to differentiate between tight and snug suturing is to pass one arm of a forceps between the suture and the conjunctiva – this can be easily performed in an eye that is snugly sutured. IOPs can then be measured again immediately following suturing to confirm there is no resulting pressure increase.
